# Evidence of Water Quality Degradation in Lower Mekong Basin Revealed by Self-Organizing Map

**DOI:** 10.1371/journal.pone.0145527

**Published:** 2016-01-05

**Authors:** Ratha Chea, Gaël Grenouillet, Sovan Lek

**Affiliations:** 1 CNRS, Université Toulouse III Paul Sabatier, ENFA, UMR5174 EDB (Laboratoire Évolution & Diversité Biologique), 118 route de Narbonne, F-31062 Toulouse, France; 2 Faculty of Agriculture and Food Processing, University of Battambang, Road 5, Battambang City, Cambodia; NSYSU, TAIWAN

## Abstract

To reach a better understanding of the spatial variability of water quality in the Lower Mekong Basin (LMB), the Self-Organizing Map (SOM) was used to classify 117 monitoring sites and hotspots of pollution within the basin identified according to water quality indicators and US-EPA guidelines. Four different clusters were identified based on their similar physicochemical characteristics. The majority of sites in upper (Laos and Thailand) and middle part (Cambodia) of the basin were grouped in two clusters, considered as good quality water with high DO and low nutrient levels. The other two clusters were mostly composed of sites in Mekong delta (Vietnam) and few sites in upstream tributaries (i.e., northwestern Thailand, Tonle Sap Lake, and swamps close to Vientiane), known for moderate to poor quality of water and characterized by high nutrient and dissolved solid levels. Overall, we found that the water in the mainstream was less polluted than its tributaries; eutrophication and salinity could be key factors affecting water quality in LMB. Moreover, the seasonal variation of water quality seemed to be less marked than spatial variation occurring along the longitudinal gradient of Mekong River. Significant degradations were mainly associated with human disturbance and particularly apparent in sites distributed along the man-made canals in Vietnam delta where population growth and agricultural development are intensive.

## Introduction

Globally, water resources are significantly threatened by various anthropogenic activities, including climate change which is particularly intense in tropical regions, and notably Asia [1,2]. As a result, many rivers in the region are grossly polluted and huge portions of their drainage basins and floodplains have been deforested or otherwise degraded [3].

The Lower Mekong Basin (LMB) water resources are extremely important for the four riparian countries downstream of China and Myanmar, i.e. Thailand, Laos, Cambodia, and Vietnam. Today, the basin serves for a variety of water-related activities—watershed management, agriculture, fisheries, navigation and transport, hydropower development, tourism and recreation, which support the livelihood of more than 60 million people living in the basin [4]. Apparently, among the largest great rivers around the world, the Mekong River is probably the largest river to feed vast numbers of people relying on it for nutritional needs (e.g. fish and other aquatic organisms) [5]. However, over the last 30 years, the Mekong River has been faced with environmental degradation due to the multiple sources of pressure, i.e. rapid population growth, industrialization, intensive agricultural development. These have left natural resource managers with a number of serious challenges regarding the preservation of biodiversity and ecosystem health. Consequently, water quality is becoming dramatically degraded from upstream to downstream in many part of the basin and evidence indicates that the diversity and productivity of freshwater species and ecosystems is also adversely affected [2,6,7]. This is of serious concern to all riparian countries since their livelihood depend mainly on the environment health and ecosystem services provided by the Mekong river and its tributaries [4]. Thus, water quality is the key factor determining the environmental health and quality of the ecosystem [8].

Recent studies on Mekong mainstream have addressed hydrological issues, sediment fluxes, climate change, and the impact of upstream dams on the Mekong’s floodplains [9–11]. However, the global perspectives on water quality patterns across the whole catchment are still questionable. Of course, under the water quality monitoring program of Mekong River Commission (MRC), the annual water quality assessment has been reported for monitoring sites along the main river and tributaries, yet the secondary sites were not well assessed. So far, there is no substantial scientific study to quantify the physicochemical characteristics of water at the whole basin scale, let alone the water quality studies conducted at local scale by each member country.

In the Mekong delta, many studies have shown surface water pollution in the man-made canals and some densely populated cities such as Chau Doc, Can Tho, My Thaun, that could threaten human, animal and ecosystem health given the fact that this water source is intensively used for drinking, irrigation and domestic services [12–14]. In Thailand, water quality monitoring by the Pollution Control Department (PCD) revealed that 68% of water bodies were suitable for use by agriculture and for general consumption being of good and moderate quality but no surface water was categorized as being of very good quality [15]. Compared to Thailand, water quality monitoring in Laos, Cambodia and Vietnam is very limited and monitoring complicated, in particular the unclear definition of responsibilities and competences among different ministries and agencies at national and regional levels [16–19]. In Laos, recent studies into water quality have shown high concentrations of nutrients (NO_3_ and P), nitrogenous matter and TSS at Vientiane city and in upstream Mekong located in northern of Laos [20,21]. In Cambodia, some concerns have been raised regarding the degradation of water quality in Tonle Sap lake and the 3S river system (Sesan, Sekong, Sraepork) [22,23]. Although multiple pressures affect the water quality in the LMB; the Mekong River Commission (MRC) reported that water quality in the main channel is still good, except for some local degradation [24–26]. As an example of water quality assessment in 2008, at least 50 monitoring sites in the basin were assessed for human health and aquatic life using dissolved oxygen, ammonium, total phosphorus, chemical oxygen demand, pH and nitrate as pollution indicators. The results indicated that 31% of all samples were rated as B (good), while 38% were rated as C (moderate) and 25% as D (poor); only 6% of samples were categorized as A (high quality) [4,26]. However, these previous studies were limited to describe and to discuss further variation of water quality patterns across the whole basin. Specifically, the parameters that could reflect the physicochemical characteristics in each compartment of the basin (i.e., upper, middle, lower part) were not well addressed, nor informed. Thus, water quality status, as well as ecological health of the basin were somehow over or underestimated. Besides, most of regular assessments were located in the main channel, while many tributaries were overlooked. Accordingly, with the challenges of population growth, urbanization, waste management and the need to feed, it is expected that the degradation of water quality in both the main channel and the tributaries of the LMB will occur.

In this context, the objective of the present work was to assess the spatial variation of water quality in the LMB based on physicochemical characteristics of the surface water. More specifically we used a Self-Organizing Map to characterize the surface water status and to identify the hotspots of pollution within the 4 riparian countries, generating a whole picture of water quality variation at the basin scale. The water quality status is helpful for defining the environmental situation and detecting the anthropogenic impact on biodiversity. This information is essential not only for people living in the basin and for the governments of the 4 countries to prioritize the management plan and action in order to reduce environmental and human health risks, but also it could help to improve the effectiveness of existing water quality monitoring programs in river basins worldwide.

## Materials and Methods

### Study area: Lower Mekong Basin

The Mekong River is the world's 12^th^ longest river and the 7^th^ longest in Asia. Its estimated length is 4,350 km and it drains an area of 795,000 km^2^, discharging 457 km^3^ of water annually. From the Tibetan Plateau this river runs through China's Yunnan province, Burma (Myanmar), Laos, Thailand, Cambodia and Vietnam. The Mekong river basin is functionally divided into two parts: the Upper Mekong basin (UMB) and the Lower Mekong basin (LMB) [27]. The UMB, located in the temperate and high altitude semi-tropical zone of China (Lancang Jiang), is covered by alpine and mountainous areas with a low population density while the LMB, located in the tropical zone of South-East Asia, drains more than 76% (60 000 km^2^) of the Mekong basin and is characterized by a low, flat topography with a high population density [20]. The LMB is well known for its rich freshwater biodiversity; particularly fish which provides a livelihood for million people [28]. The hydrology of the LMB is characterized by two monsoons, from May to October and from November to March, with the former bringing most of the annual rain. The water level begins rising in May and peaks in September reaching an average flow of 45 000 m^3^/s [27].

In Cambodia, Tonle Sap Lake, which is the largest freshwater lake in Southeast Asia [23], is connected to the Mekong through the Tonle Sap River. This creates an exceptional hydrological cycle; during the rainy seasons the excess water from Mekong river enters the Tonle Sap lake, expanding the area it covers from 2 500 km^2^ to 15 000 km^2^ and creating an extensive wetland around the entire lake. When the rain ceases and water levels drop in the Mekong, reverse flow drains the lake which flows into the Mekong delta [29]. The Mekong delta is known for its high density of artificial canals which are for domestic and agricultural utilization, connected to Bassac and Mekong rivers [30]. During the wet season, 35–50% of the total surface area of the delta is flooded [31]. Sea-water intrusion dominates the hydrology along the coastal areas with water level fluctuations of more than 3 m due to the tidal regime [14]. The reverse flow from Tonle Sap is very important to balance the Mekong delta in Vietnam during the dry season ensuring freshwater flow into the Mekong delta and protecting against sea water intrusion.

### Monitoring sites

**Fig 1:**
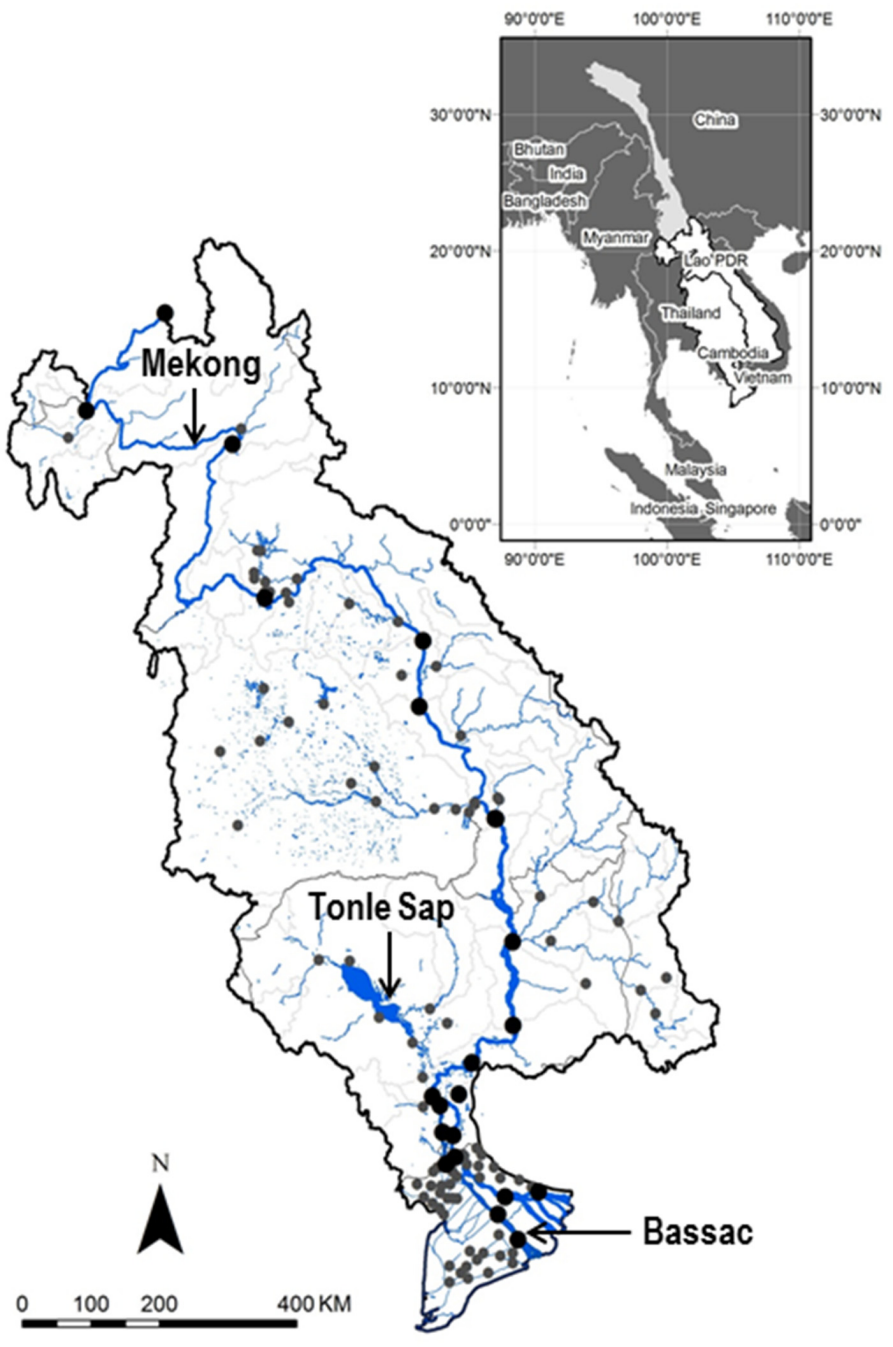
Lower Mekong Basin and monitoring site localities (grey dots for tributaries and big black dots for Main River).

The data used were provided by the Mekong River Commission (MRC), under the Water Quality Monitoring Network Program. The monitoring program has begun in 1985 in Laos-Vietnam-Thailand and in 1995 in Cambodia [4]. So far there are a total of 132 monitoring sites including 33 newer sites established in the 2000s and 99 older sites established before 1995. To study the spatial variation of water quality in the LMB, we focused on 117 monitoring sites (Fig 1), among which 22 were located in Cambodia, 25 in Laos, 20 in Thailand, and 50 in Vietnam. Sixteen physicochemical variables (Table 1) were selected from the 46 variables in the dataset for water quality analysis. The selection was based also on the physicochemical importance of variables in explaining the water quality status and data completeness criteria (i.e. variables measured at all sites, with less than 30% missing values). The water quality data used in this study included 24,383 samples monitored from 1985 to 2010 at 117 sites with a monthly time scale base.

**Table 1:** Physicochemical variables and measurement unit of selected variables for water quality analysis.

Parameters	Units	Parameters	Units
Water temperature (T)	°C	Magnesium (Mg^2+^)	mgL^-1^
Conductivity (EC)	μScm^-1^	Chloride (Cl^-^)	mgL^-1^
Total suspended solids (TSS)	mgL^-1^	Sulphate (SO_4_^2-^)	mgL^-1^
pH	SU	Alkalinity as HCO_3_^-^	mgL^-1^
Dissolved oxygen (DO)	mgL^-1^	Nitrate (NO_3_^-^)	mgL^-1^
Sodium (Na^+^)	mgL^-1^	Total phosphorus (TP)	mgL^-1^
Potassium (K^+^)	mgL^-1^	Chemical oxygen demand (COD_mn_)	mgL^-1^
Calcium (Ca^2+^)	mgL^-1^	Total ammonia (NH_3_^-^ + NH_4_^+^)	mgL^-1^

### Data analysis and modelling approach

#### Overview

First, annual averages of each variable were calculated at each site. Then the medians of the annual means were computed to describe the physicochemical characteristics at each site over the study period. Only median values were used for the analysis as they are considered as a robust statistical indicator minimizing the effects of noise in the dataset that can influence analysis [32]. Unsupervised artificial neural networks (Self-Organizing Maps) were then used to classify the monitoring sites into different clusters based on the similarities of samples defined by the 16 physicochemical variables. Last, water quality assessment was compiled to identify pollution hotspots within the basin according to the selected water quality indicators and US-EPA guideline [33].

#### Self-Organizing Map (SOM)

The SOM is a non-supervised artificial neural network that is trained using a competitive learning process to produce a two-dimensional representation of the training samples, called a map [34]. The SOM is a powerful method to classify complex data ruled by non-linear relationships. Recent applications of SOM in water quality and aquatic ecology have yielded very good results in patterning and prediction [35–38]. Mostly, the SOM consists of 2 main types of layers: the input layer and the output layer. In the present case, the input layer contained 16 neurons connected to 117 training samples and the output layer is represented by the rectangular grid or map with *l* rows and *m* columns of neurons, laid out in a hexagonal lattice. During the training process, the activation level, which is the Euclidean distance between the input vectors of the 117 training samples and weight vector of each neuron on the map was calculated as follows [34]: $∥{\text{w}}^{\text{j}}-\text{x}∥=\sqrt{\sum_{\text{i}=0}^{\text{n}}{\left({\text{w}}_{\text{i}}^{\text{j}}-{\text{x}}_{\text{i}}\right)}^{2}}$, where x is the input vector or training samples described by a set of descriptors known as the physicochemical variables, *i* is the number of training samples from 1 to 117, *j* is the neuron number and w^j^ is the weight vector associated to neuron *j* on the map. With the different magnitudes of the input vector x, the activation level was calculated once the logarithmic normalization of median values of each of the 16 physicochemical variables for the studied sites was computed, and the calculation was done for each input pattern and all the neurons presented on the map.

After all sample vectors have been trained, the algorithm automatically classifies the trained samples on the map in what is known as the mapping process. The map consists of several neurons which store the weight vector as mentioned above. Normally, the classification is done through activation levels between the input and output spaces to find the winning neurons or best matching units (BMU). The neuron whose weight vector closely matches the input vector will have a small activation level, while the neuron with a weight vector very different from the input vector will exhibit the large activation level. The neuron with the smallest activation level is considered to be the winner or the BMU for the current input vector. The mapping process is continued until a stopping criterion is met, usually when the BMU is determined with the corresponding neurons of the map after completing a certain number of iterations. The algorithm was run through the SOM package in Matlab program (http://www.cis.hut.fi/somtoolbox/).

Different map sizes were tested during the training process based on a formula:$\text{c}=5\sqrt{\text{n}}$ proposed by the Laboratory of Computer and Information Science (CIS)—University of Helsinki, where *c* is the number of neurons and *n* is the number of training samples, according to the optimal values of quantization and topographic errors. Therefore, the input layer was composed of 16 neurons connected to 117 training samples and the output layer comprised 56 neurons organized in an array with 8 rows and 7 columns. Moreover, in order to define the boundaries of possible clusters in the SOM map, a hierarchical clustering on the final SOM weight vectors was used to classify SOM neurons according to Ward’s method. Kruskal-Wallis analysis was used to test the significance of the clusters identified.

### Water quality assessment

In the LMB, no specific or standard guidelines have been found in particular besides the guidelines used by MRC [4,26,39]. In 2007, MRC conducted a diagnostic study in LMB by using SEQ-Eau, i.e. Water quality evaluation system for surface water [40], evaluation system to quantify the quality status of water in main sub-basins [26]. SEQ-Eau was developed for European rivers; thus the criteria of certain parameters seem far-reaching the standards of tropical ones. Consequently, most of the evaluated sites were graded to good and very good for many parameters. Subsequently, MRC adopted new water quality guidelines based on the data set from 1985 to 2000 by using 6 indicators to evaluate the impacts of water quality on aquatic life (i.e., DO, pH, total ammonia, conductivity, total nitrate and total phosphorus) and 2 indicators for human impact (i.e., ammonium and COD) [4].

In our study, water quality assessment was undertaken based on the Environmental Protection Agency of United States (US-EPA)’ guidelines for the impact on aquatic life [33]. These guidelines were developed under the Water Clean Act 1972 and known as the foundation of water quality-based pollution control program to protect the human health and aquatic life. The guidelines consist of many criteria with specific uses (i.e., aquatic life, human, recreation, reuse, irrigation) and are potentially applicable to any kinds of water bodies (e.g., cold, cool and warm water) [41]. On the other hand, the threshold values were matched to MRC’s guidelines for the impact on aquatic life [4]. Dissolved oxygen, total phosphorus, total ammonia and chloride were used as water quality indicators to detect the surface water pollution in the LMB that could affect the river health. Numerous studies have confirmed that DO, TP, nitrates and total ammonia are the primary important parameters revealing the ecological health of the surface water [42]. Low DO can affect aquatic communities, while excessive amounts of TP, nitrates and ammonia are likely to cause significant changes in ecosystem functioning since they are very toxic and have adverse effects on human health and organisms [43]. In neutral pH values most of the ammonia in river water is in the form of NH_4_^+^ [26]. Additionally, chloride is recommended by US-EPA for salinity assessment instead of conductivity since the diurnal fluctuation of Cl^-1^ is less significant [44]. Therefore, the four selected indicators could be optimal to quantify the quality status of water and pollution hotspots in the basin. Accordingly, the assessment was done through these 4 criteria (Table 2), if the assessed site meets all criteria it would get 4 points and would be classified as Very Good water quality, as Good for 3 points, as Fair for 2 points and as Poor for 1 or 0 point.

**Table 2:** Criteria of water quality indicators used for water quality assessment with threshold values (US-EPA, 1976).

Parameter	Threshold value
Dissolved oxygen	> 5 mgL^-1^
Total phosphorus	< 50 μgL^-1^
Total ammonia	< 20 μgL^-1^
Chloride	< 250 mgL^-1^

## Results

### Water quality patterns

#### Cluster identification

**Fig 2:**
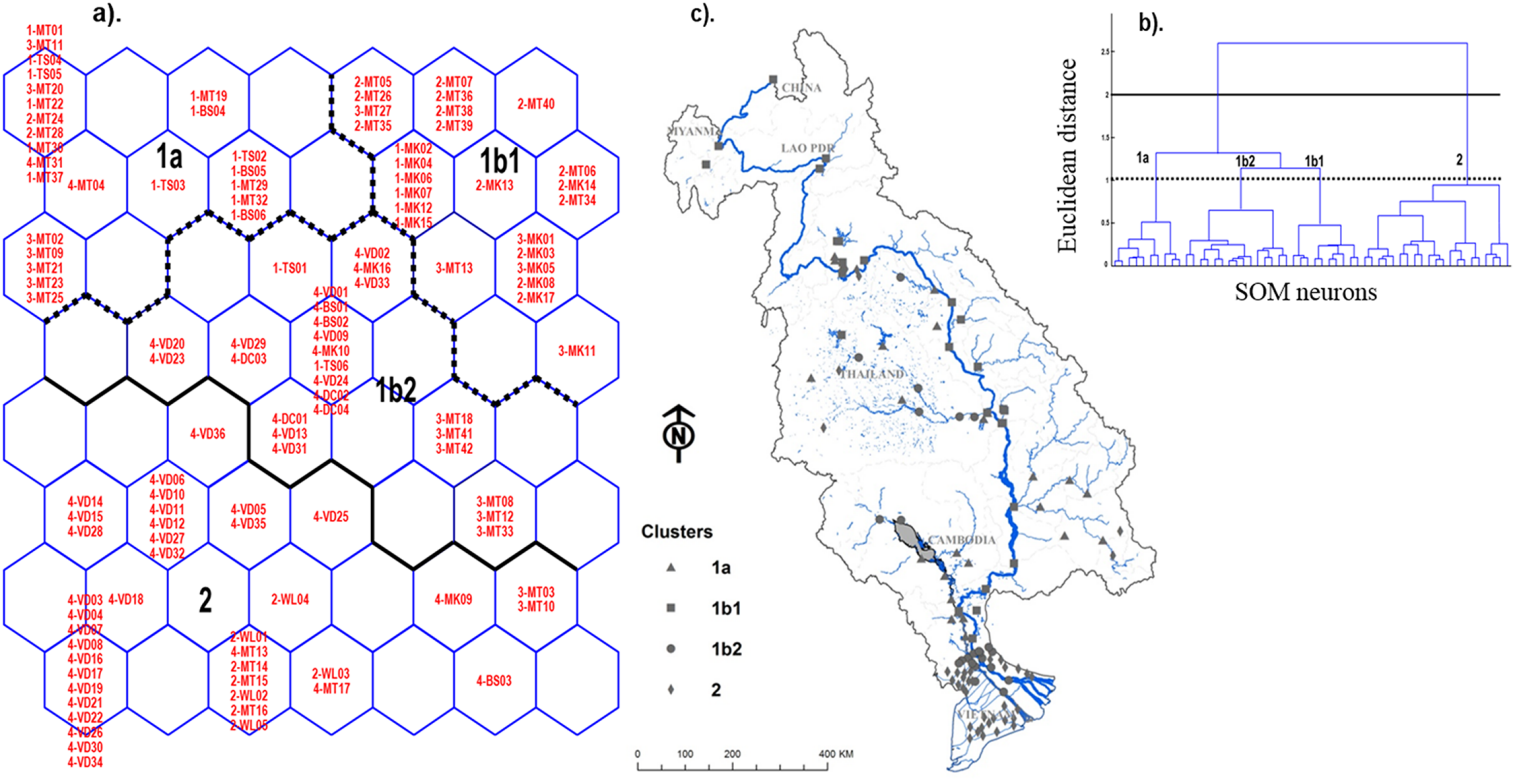
SOM results. a). Classification of monitoring sites based on their similarities from physicochemical variables on SOM output layer. b). Hierarchical clustering according to the similarity between SOM neurons. c). Map of the clustering sites in the LMB. The acronyms in the hexagonal neurons represent the monitoring sites. The sample code is composed of 5 characters; the first character is a number from 1 to 4 indicating the country code: 1 for Cambodia, 2 for Laos, 3 for Thailand and 4 for Vietnam. The rest indicates the water body type and number of sites along this water body (i.e. MK-Mekong river, BS: Bassac river, MT: Mekong tributary, VD: Vietnam delta, DC: Delta canal, TS: Tonle Sap lake, WL: Swamp).

The monitoring sites were first classified on the SOM map according to the similarity of their physicochemical characteristics (Fig 2a). Different map sizes were tested and the optimum map size compromised 56 neurons [8 x 7] with the minimum value of quantisation (0.34) and topographic errors (0.009).

The similarity of neurons allowed us to divide the SOM map into 4 clusters (Fig 2b): 2 main clusters 1 & 2 with sub-groups (1a, 1b1, 1b2). This distribution pattern revealed the longitudinal water system of the Mekong in the LMB between upstream LMB (cluster 1) and Mekong delta (cluster 2). Cluster 1a included 25 sites extending through the floodplain of the Mekong River, in the middle part of the LMB between Cambodia and the southern part of Thailand. This group of sites was distributed along the larger Mekong tributaries such as the 3S Rivers (Sraepork, Sesan, Sekong) and Tonle Sap River connecting the Tonle Sap Lake to the Mekong (Fig 2c). Cluster 1b1 was defined by 26 sites mostly in the Mekong main channel from the head in Laos to the delta at the border between Cambodia and Vietnam. Some sites in this group were located in tributaries of the Mekong in Laos (Fig 2c). Cluster 1b2 was determined by 26 sites located in the transitional zone between the Lower Mekong River and its delta. Most of the sites were distributed along the 2 main branches of the Mekong (Mekong and Bassac rivers) closed to the border of Cambodia and Vietnam. Only a few sites of this cluster were found in the delta (i.e., Chau Doc, Can Tho, My Thuan) and upstream of Mun river in Thailand (Fig 2c). Cluster 2 grouped 40 sites, the majority of which were located in the Vietnam delta, along the artificial canals that connect to Bassac and Mekong rivers next to the outlet of the river, known to be brackish zones (Fig 2c). The sites in this group were generally affected by the tides with an average depth of 0.3 to 0.7 m [45]. A few sites in this cluster were found in Laos’ swamps and tributaries close to Vientiane and in the upstream part of the 3S rivers located in Vietnam.

#### Cluster interpretation

**Fig 3:**
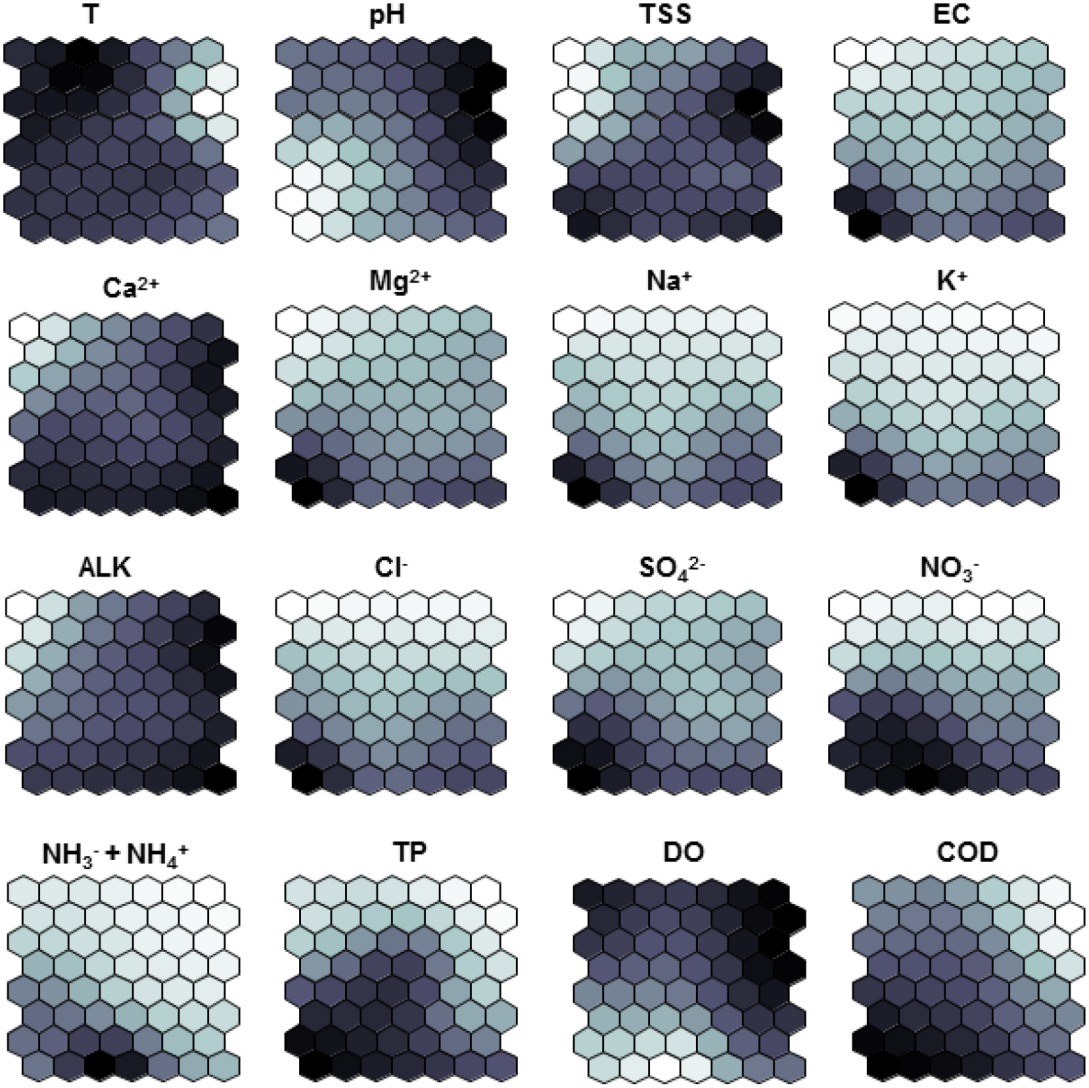
SOM component map showing the contribution of 16 variables on the SOM model. Dark areas represent high values of each input variable.

**Fig 4:**
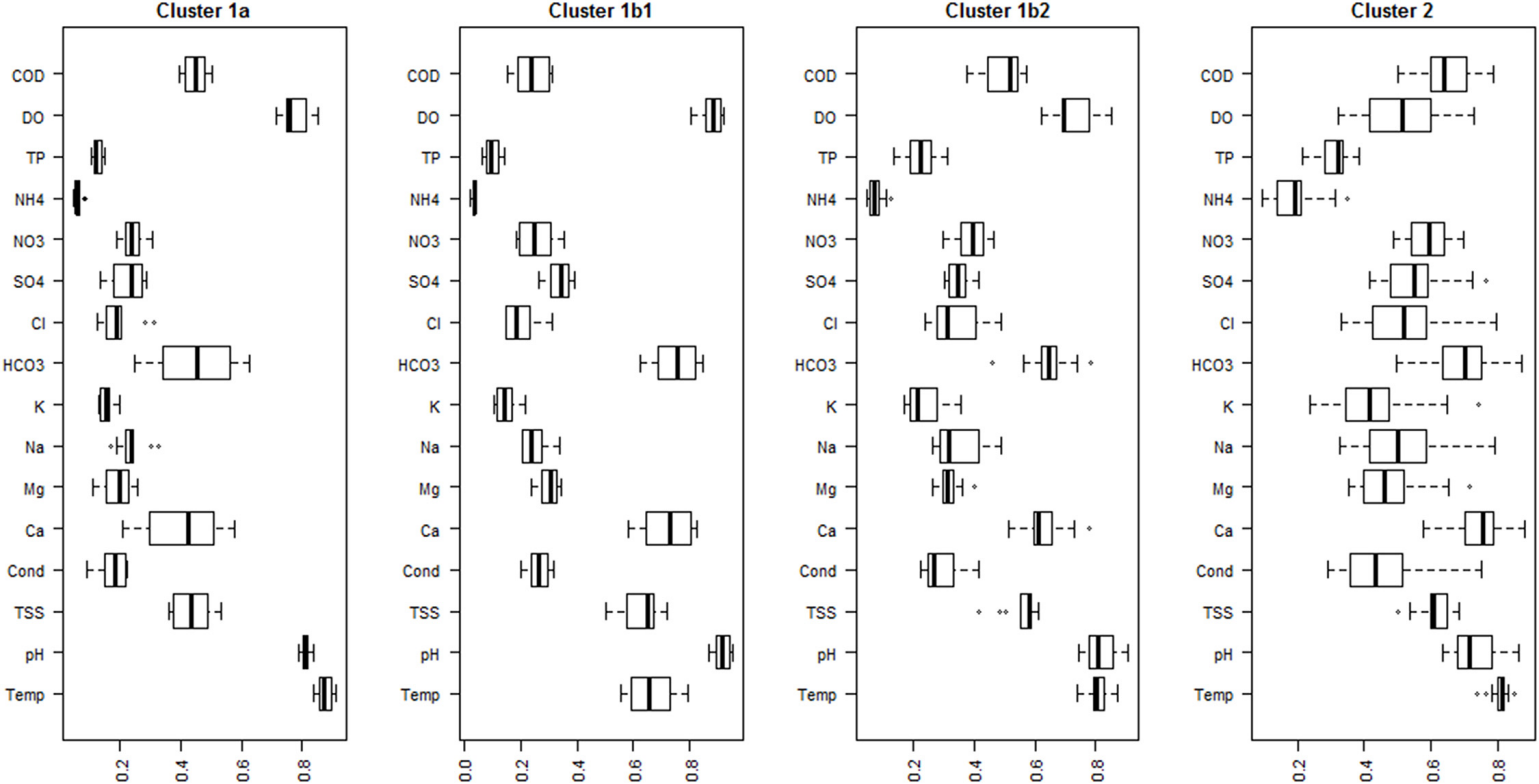
Boxplot of weight vectors (codebook) between SOM neurons. The boxplots represent 25, 50 (median) and 75 percentiles of Euclidean distance between SOM neurons, while the whiskers indicate 10 and 90 percentiles. Large boxes indicate large variations between neighbourhood neurons of each cluster, while high values for the parameter in each plot denotes its important contribution to all neurons associated to the clusters.

The SOM component map was constructed to visualise the contribution of each physicochemical parameter to the map (Fig 3). The weight vectors of the SOM neurons revealed the influence of each physicochemical variable in the characterization of the identified clusters (Fig 4). Cluster 1a was mainly characterized by high water temperature, relatively high dissolved oxygen and pH, comparatively low levels of TSS and all cations and anions (i.e. Ca^2+^, Mg^2+^, Na^+^, Cl^-^, K^+^ and SO_4_^-2^) i.e. dissolved solids in the water. Cluster 1b1 was determined by high levels of oxygen and high pH, followed by low water temperature and TSS values, and high level of alkalinity and calcium. Cluster 1b2 had similar characteristics to cluster 1a, it was categorized by high temperature, slightly reduced oxygen and pH values, and relatively increased TSS, calcium and alkalinity. Completely different from the previous clusters, cluster 2 was primarily determined by high values of nutrients (nitrates, total phosphorus and total ammonia) and all dissolved solids. Moreover, the strong variations were noted in nutrients and ions between the monitoring sites of cluster 2.

### Water quality assessment

In cluster 1a, 13 sites were categorized as being of good quality while 12 were fair (Table 3). Fourteen sites of cluster 1b1 were graded as good quality and the rest were assessed as fair quality. In both, clusters 1a and 1b1 more than half the sites were ordered as good. In cluster 1b2, most of the sites (23 sites) were classified as fair, while 2 sites were good and 1 was poor. The water quality ranged from poor to fair in cluster 2, more than 60% of sites (24 sites) were typed as poor and 16 considered as fair. In the whole basin, 29, 63 and 25 sites were found as good, fair, and poor quality, respectively, while no sites were categorized as very good quality (Table 3).

**Table 3:** Water quality assessment based on the 4 indicators.

	AVery Good	BGood	CFair	DPoor
**Cluster 1a**	0	13	12	0
**Cluster 1b1**	0	14	12	0
**Cluster 1b2**	0	2	23	1
**Cluster 2**	0	0	16	24

**Fig 5:**
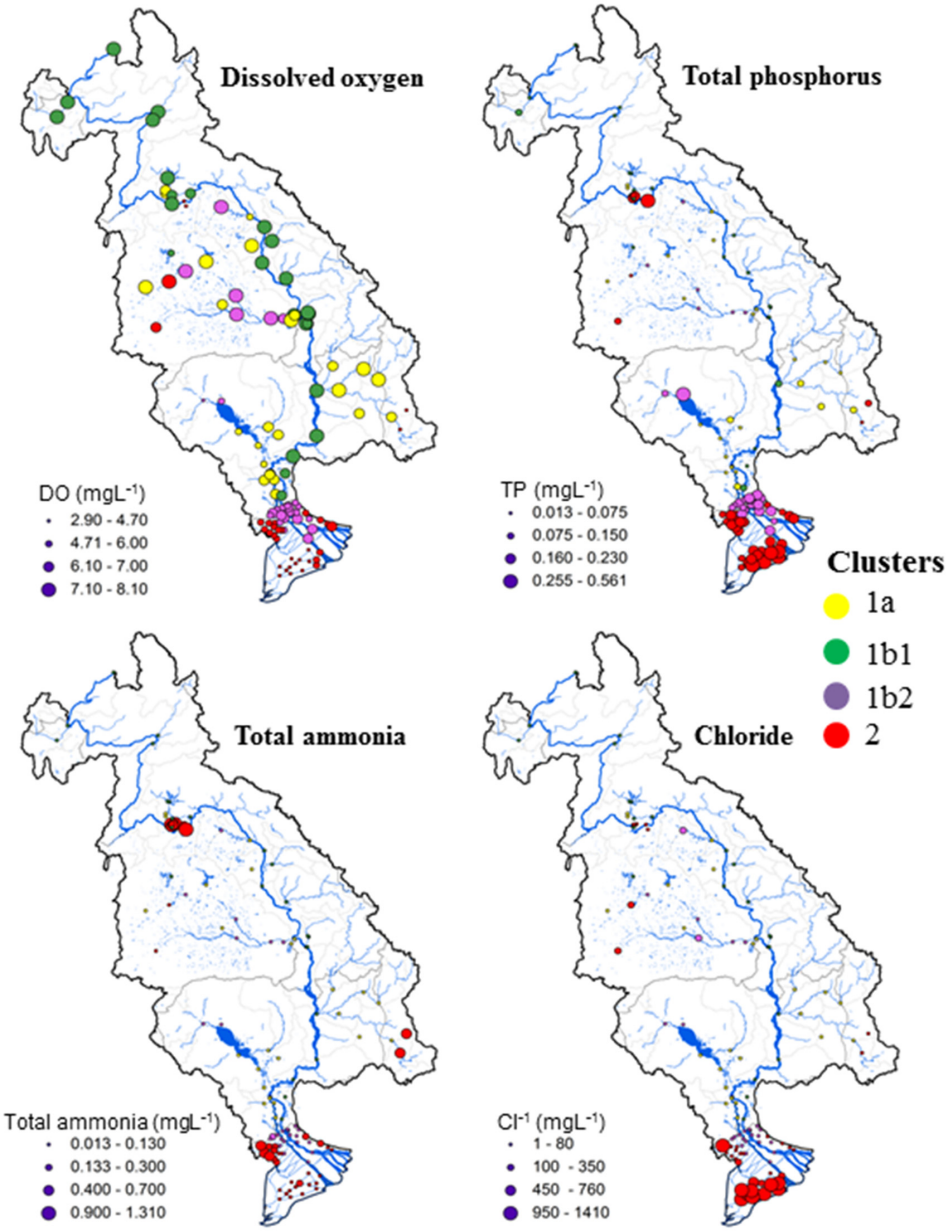
Geographic representation of annual median concentration of dissolved oxygen, total phosphorus, total ammonia, and chloride in LMB.

**Fig 6:**
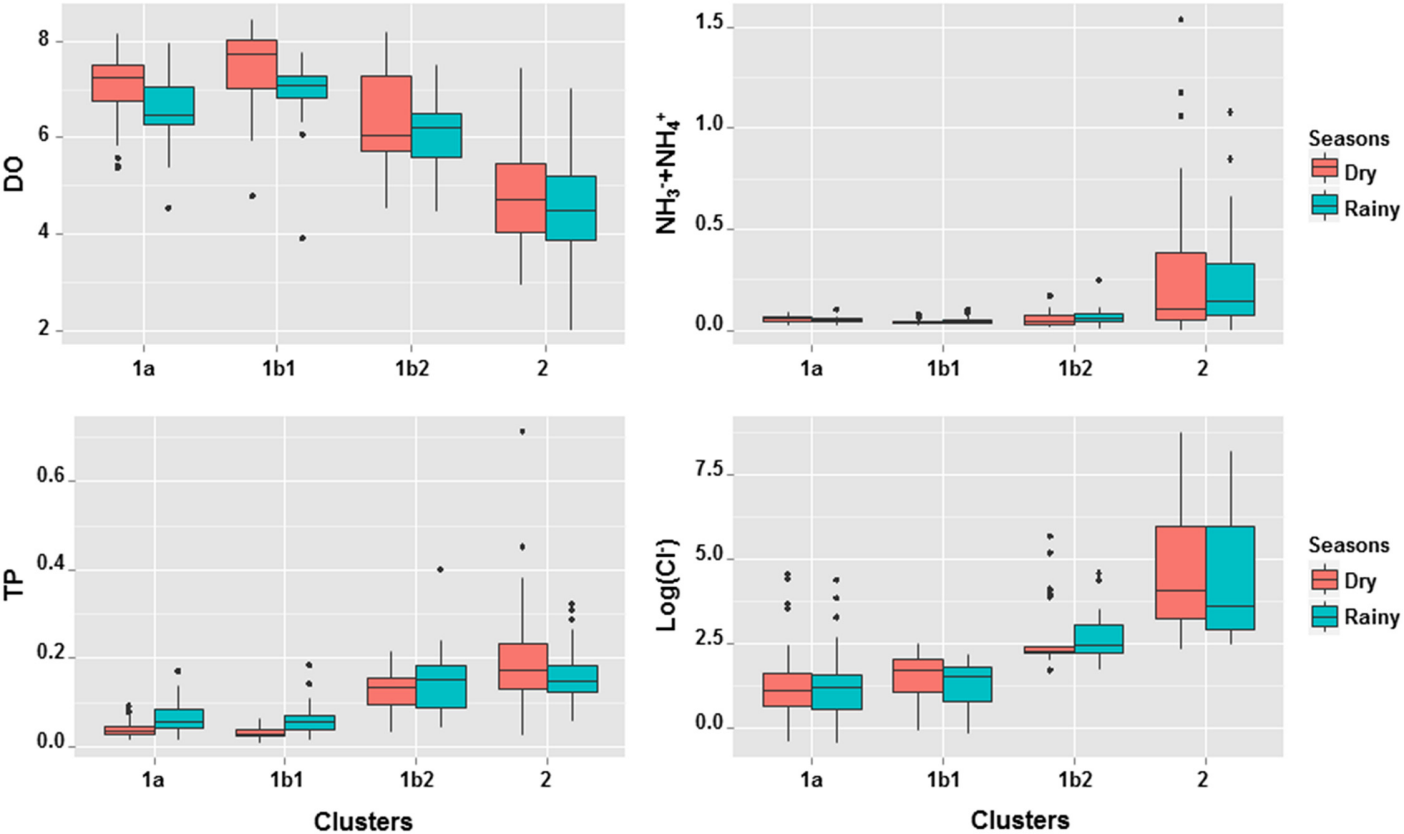
Seasonal variations between the dry (in red) and rainy (in blue) seasons for dissolved oxygen (DO), total phosphorus (TP), total ammonia (NH_3_^-^+NH_4_^+^) and chloride (Cl^-^) within the four identified clusters. Vertical axis indicates the concentration of DO, TP, total ammonia and Cl^-^ in logarithmic scale in mgL^-1^.

Spatial patterns in water quality in LMB were observed in the geographic representation of annual concentration variation of the four water quality indicators at each site in the basin (Fig 5). The gradient of water quality was seen to vary significantly between upstream of the LMB and its delta. Indeed, the DO started to decline in the delta while the phosphorus concentration increased. The same patterns of DO and TP were also observed around Tonle Sap Lake in Cambodia. Regarding chloride, high concentrations were found for all coastal sites in the Vietnam delta and moderately high concentrations at the head of Mun river (Khorat plateau) in Thailand. Meanwhile, the high concentration of total ammonia was patterned particularly in swamps located close to Vientiane city in Laos and relatively for many sites close to the Cambodian border and the head of the 3S Rivers in Vietnam. Overall, these spatial patterns were consistent among dry and rainy seasons, since no differences were observed between the two seasons (Kruskal-Wallis test, p>0.05) (Fig 6).

## Discussion

### Spatial patterns in water characteristics in LMB

Variability of water characteristics in LMB was defined by different physical and chemical characteristics in clusters 1a, 1b1, 1b2 and 2. In our study, cluster 2 was completely different from other clusters; it characterized the Mekong delta, which was mainly affected by multiple point and non-point sources of pollutions. Recent studies of water quality in the Mekong delta have shown high levels of pollution in terms of organic pollutants, salts, metals and microorganisms that could threaten human, animal and ecosystem health [14,46]. Our study revealed that the nutrient concentration was highest mainly along the delta canals, especially the concentration of TP which ranged from 0.13 mgL^-1^ in cluster 1b2 to 0.56 mgL^-1^ in cluster 2. Previous studied have concluded that the pollution from Can Tho city could affect human health within the delta [12,47]. Besides, pH and DO were depleted for most of the sites due to the decomposition of organic matter and storm runoff affected by land use in acid sulfate soils [48,49]. Furthermore, the increase of acid in the water could result from the intensive aquaculture in the delta canals and chemical fertilizers from agriculture. Among the adverse effects of shrimp farming are low pH values and acidification due to the release of feed chemical elements [50]. Associated with many chemical reactions in the water (e.g. the relationship between total ammonia and ammonium), acidification could thus be one of main issues in the delta likely to affect biodiversity. In parallel, our study illustrated the degradation of water quality in swamps close to Vientiane city in Laos. Gerrard (2004) reported the effect of Vientiane municipal waste on the swamps (That Luang basin connecting to Mekong) affecting environmental health [51,52]. In Vientiane, direct discharge of municipal waste into the swamps can be one of the reasons leading to excess concentrations of total ammonia. The concentration of total ammonia ranged from 0.013 mgL^-1^ to 1.3 mgL^-1^, with maximum values (i.e. greater than 1 mgL^-1^) for all the sites in the swamps of Laos. In the delta, stream farming and catfish aquaculture also contributed to the excess levels of ammonium in the water body [53]. Apart from this, the daily effects of sea water intrusion drive the salinity increase in the delta, especially in the coastal zone of Mekong delta (Bassac river) the concentration of salt at certain sites attaining 1400 mgL^-1^ chloride with similar patterns for conductivity (22 000 μScm^-1^). Moreover, TSS were found to be high for most of the sites in the delta, and could be accounted for firstly by the accumulated sediments from the upstream Mekong and secondly by erosion within the delta zone due to the intensive agricultural activities. Therefore, domestic waste from the municipalities, agriculture, and seawater intrusion remain the main sources of pollution in the delta.

In freshwater systems, dissolved solids consist of inorganic salts, small amounts of organic matter and other dissolved material [54] are CO_3_^-^, Ca^2+^, Mg^2+^, Na^+^, Cl^-^, K^+^ and SO_4_^-2^. Strong correlations between conductivity and dissolved solids were found for all monitored sites in the LMB. In contrast to cluster 2 and 1b2, alkalinity and concentrations of dissolved solids were found to be low in cluster 1a and 1b1. In the transitional zone between the Mekong and its delta (cluster 1b2), the nutrients started to increase and reached a maximum for certain sites along the delta canals, especially phosphorus which is a toxic element and subject to bioaccumulation. Excess concentrations of TP can cause biological disturbances such as eutrophication. These could result from the direct municipal discharges and urban storm water runoff from the densely populated cities (i.e. Vientiane, Phnom Penh, Can Tho, Chau Doc) and also agricultural activities (i.e. shrimp farming, catfish farming in Vietnam delta) which are the main source of pollution [55]. On the other hand, the concentration of TP in the main channel was lower than in the swamps and delta canals.

In Mekong mainstream (cluster 1b1), the water temperature increased from 20°C to 30°C from upstream to downstream. The lowest temperature was found in a few high altitude sites located in Laos and Thailand, while high temperatures were observed in the middle of the basin (cluster 1a), particularly around Tonle Sap Lake and along the Bassac river where the mean temperature was about 30°C. Moreover, the sediment load in Mekong mainstream seems to be much greater than in its tributaries and floodplains [4]. As a result, the annual median concentration of TSS in main channel reached maximum for certain upstream sites in Laos and Thailand, from Loung Prahban to Nakhon Phnom, comparatively 10 to 20 times higher than in tributaries and floodplains (e.g., Tonle Sap Lake and 3S rivers). This could be a cause for concern since the sediment has important implications in the river system and is a crucial element in aquatic systems, fisheries, agriculture, water supply and navigation [56]. Recent studies on sediment transport showed that 84% of sediments from the Mekong are retained in the lake and less than 15% contribute to Vietnam delta sediment [57]. This could explain the siltation at the bottom of the lake and in the floodplains. Downstream of Phnom Penh, capitals of Cambodia, oxygen levels started to decline and were found to be limited on arriving in the delta, potentially affected by agricultural, domestic and industrial disposal. This could also result from accumulation of organic matter from discharge from Phnom Penh city and two other big cities in the delta (Chau Doc and Can Tho), excessive algae growth caused by phosphorus and decomposition of submerged plants. In the main channel, total ammonia was found to be less than in its tributaries.

### Water quality degradation

The variation in water characteristics in clusters 1 and 2 could reveal the water quality changes along the basin, a significant decline observed in the Mekong delta and its transitional zone. From Laos to Thailand (cluster 1b1, upstream), the water quality was found to be good except the degradation in some sections of the mainstream such as in Chiang Sean, Vientiane and Nakhon Phanom. After connecting with Mun river in Thailand, the water quality declined to fair, continuously in Khong Chiam, Pakse, Stung Treng. After leaving the Khone falls and before connecting with Tonle Sap river, the water quality was found to be good at Kratie. In Cambodia, the water quality started to decline again, especially below the capital Phnom Penh. The water quality was degraded across the border along the densely populated cities of Vietnam delta Chau Doc, Than Chau, Can Tho, My Tho, and Dai Ngai. In the delta, the water quality became dramatically degraded, particularly in the coastal zone sites.

The spatial trends in water quality degradation can be clearly observed on the maps of water quality indicators in Fig 5. Apparently, the sharp decline of DO and significant increase of TP in delta region, north Tonle Sap and tributaries below Vientiane were associated mainly to anthropogenic activities rather than natural processes, as illustrated in S1 Fig. Upstream (above Vientiane), the trends of DO and TP varied gradually, while significant shifts were noticed for sites below the densely populated cities (i.e., Vientiane, Phnom Penh, Chau Doc, Tan Chau) (S1 Fig). The condition was drastic for tributaries sites below Vientiane, as well as below Phnom Penh city and along the man-made canals in delta, where TP and DO showed rough shifts. The peak of TP and drop of DO indicated clearly significant impacts on water quality due to human activities. According to recent study on nutrient dynamics, 75% of TP in LMB originate from intensive agricultural activities, particularly the uses of fertilizers [24]. Besides, we found very high concentrations of total ammonia in most of the monitoring sites, especially in the Laos’s swamps and delta canals (Fig 5; S1 Fig). Otherwise, the excess concentration of ammonium and the increase of phosphorus in some areas of the basin (e.g., north of Tonle Sap Lake in Cambodia and the Khorat plateau upstream of the Mun river in Thailand) could also be a concern with organic pollution leading to eutrophication and salinity increase. So far, Sdivong and Teng (2006) have drawn their concerns on the increase of nutrients in Mekong, as well as the chemical pollution originating from agricultural run-off and pesticides, affecting Mekong delta, Tonle Sap Lake and north-eastern tributaries of Thailand [58]. Feda et al. (2004) confirmed the rapid growth of algal production leading to eutrophication in these regions, potentially during dry season when the input of TP is excessive [59]. Furthermore, the salinity increase in the basin would be also the concern in LMB. Apparently, chloride seems to be stable in main channel and suddenly increased at delta canals (S1 Fig). In northwestern part of Thailand (Khorat plateau), salinity increase was noticed as well, as reported in MRC (2008) (Fig 5) [4]. Mainly, the effects of salinity in the basin were linked to both natural process (i.e., sea water intrusion in the delta region) and human activities (i.e., agriculture, shrimp farming, fish aquaculture).

Nevertheless, compared to other Asian rivers (e.g., Yangtze, Yellow, Ganges), Mekong was considered as pristine river [60]. According to our assessment, the water quality in the LMB ranged from fair to good, apart from the delta, and Mekong mainstream was considered to be less polluted than its tributaries. Overall, we found good agreement between our assessment and MRC water quality monitoring reports; the differences could result from the water quality indicators and the guidelines used for the assessment [4,15]. According to our understanding, the degradation of water quality in main channel for some sections can be caused by the low quality inputs from tributaries. Consequently, the self-cleaning mechanisms of running water in the mainstream may control cross-border pollution transfer [61].

Under the influence of Monsoon, the significant seasonal changes of flow could induce the variation in water quality. However, according to our result, there was no significant difference of water quality between the 2 seasons at the basin scale (Fig 6). So far, Liljeström et al. (2012) showed that TP slightly increased during rainy season and peak in July-September; while Hart et al. (2001) reported the lowest DO and highest TP values in lower part of the basin during dry season [24,62]. Indeed, the seasonal change reported would mainly reflect the nutrient dynamics within the main channel; while at the basin scale, the seasonal effect seem to be less important than the spatial variation occurring among compartments of the basin (i.e., tributaries, wetland and main river). Consequently, we found that the spatial variations of water quality along the longitudinal gradient of LMB significantly exceeded the seasonal variations. Previous study has also demonstrated the weak negative relationship between water discharge and physicochemical parameters in LMB, except for TSS and conductivity which were positively correlated to river discharge [63]. Such seasonal effects of hydrological regimes could be more apparent for local impacts on water quality in tributaries rather than in mainstream, and could reflect different water quality dynamics between main channel and tributaries.

### Pollution hotspots in LMB

In LMB, nitrates seem to be less harmful than phosphorus, with concentrations below or slightly above the thresholds. Mekong delta, Tonle Sap Lake, the swamps close to Vientiane, Khorat plateau could be considered as pollution hotspots. The sharp degradation of water quality in these regions was associated mainly to agricultural development, urbanization and industrial waste. In the coastal area of the Mekong delta in Vietnam, the water quality was very poor due to the multiple impacts from upstream in combination with the effect of sea water intrusion. Many studies have identified the degradation of water quality in the canals built in the Mekong delta [13,14,64,65]. Consequently, the pollution will become exacerbated in these areas under the intensive stress of population growth, industrialization, agriculture and tourism, which require food production and economic growth. These would be the major concerns for the riparian countries [6,24,58,66]. Moreover, these impacts could be also associated with the hydropower development in upstream Mekong [67]. Yet, our study was limited to conclude on the impacts of dams on water quality. To date, there is no dam across the main channel in LMB; while 5 of 8 planned dams were operating in upper Mekong basin in China. Previous studies on the impacts of Chinese dams on LMB have raised major issues concerning sediment trapping in dam reservoirs, flow regime regulation, habitat and biodiversity losses and food security in the basin [66,68]. Yet, there is no evidence of these impacts on water quality in LMB, as well as impacts of tributary dams [69]. While the contamination of heavy metals (Zn, Cr, Cu, Pb, As) in upstream basin has been related to the impacts of Chinese dams [70], further studies are clearly needed. In this context, we could expect that in the coming years, the effect of water quality might intensify since Laos, Thailand, and Cambodia propose to construct more dams across the main channel (e.g., 11 planned dams) and across large tributaries for electricity and agriculture purposes [71].

## Conclusion

With the challenges of population growth, urbanization, wastewater management, the need to feed of people, and the need for exports, changes in water quality in the LMB are expected. However, in spite of the multiple pressures from point and non-point sources of pollution, this study showed that the Mekong mainstream was less polluted than its tributaries. Potentially, the degradation of water quality in the mainstream was caused by the low quality of water discharged from tributaries. Eutrophication and salinity increases in many tributaries could represent the main water quality issues in the LMB, particularly in areas identified as pollution hotspots.

To date, few studies have quantified the impact of eutrophication on aquatic life (e.g. fish and fisheries resources) in LMB, although this concern has been evoked. Consistently, we raise the major concern on eutrophication and salinity increase in pollution hotspots, where urgent effective management is needed to mitigate these impacts. Indeed, as in the delta, to feed the population and enable economic growth, agriculture and aquaculture are prioritized for people living there. In the meantime, positive signs can be seen at national and regional levels as water quality monitoring programs and basin management plans have been implemented to improve the water quality and biodiversity. Improving water quality is another challenge for riparian countries; they would have strong support and participation from the people and governments of the four nations living in the basin.

To conclude, our study suggests that the monitoring efforts should be consolidated more for the group of sites in clusters 1b2 and 2, and particularly all pollution hotspot zones. Biological parameters such as Chlorophyll as well as heavy metals should also be monitored regularly. In perspective, the study of temporal dynamics of water quality associated with the influence of dams under different scenarios could be substantial to enhance the effectiveness of the existing monitoring programs and future management plans in the basin.

## Supporting Information

S1 Fig Variations in dissolved oxygen (DO), total phosphorus (TP), total ammonia (NH_3_^-^+NH_4_^+^) and chloride (Cl^-^) along the longitudinal gradient (i.e., distance from the sea in km) for Mekong River (Circle lines) and its tributaries (dot lines). Vertical axis indicates the annual median values of concentration of DO, TP, total ammonia and Cl^-^ in logarithmic scale (mgL^-1^).
(TIF)
