# Supplementary figures and images for: Evidence of Water Quality Degradation in Lower Mekong Basin Revealed by Self-Organizing Map
Source: PLoS One. 2016 Jan 5;11(1):e0145527. doi: 10.1371/journal.pone.0145527 (PMC4701190; doi:10.1371/journal.pone.0145527)

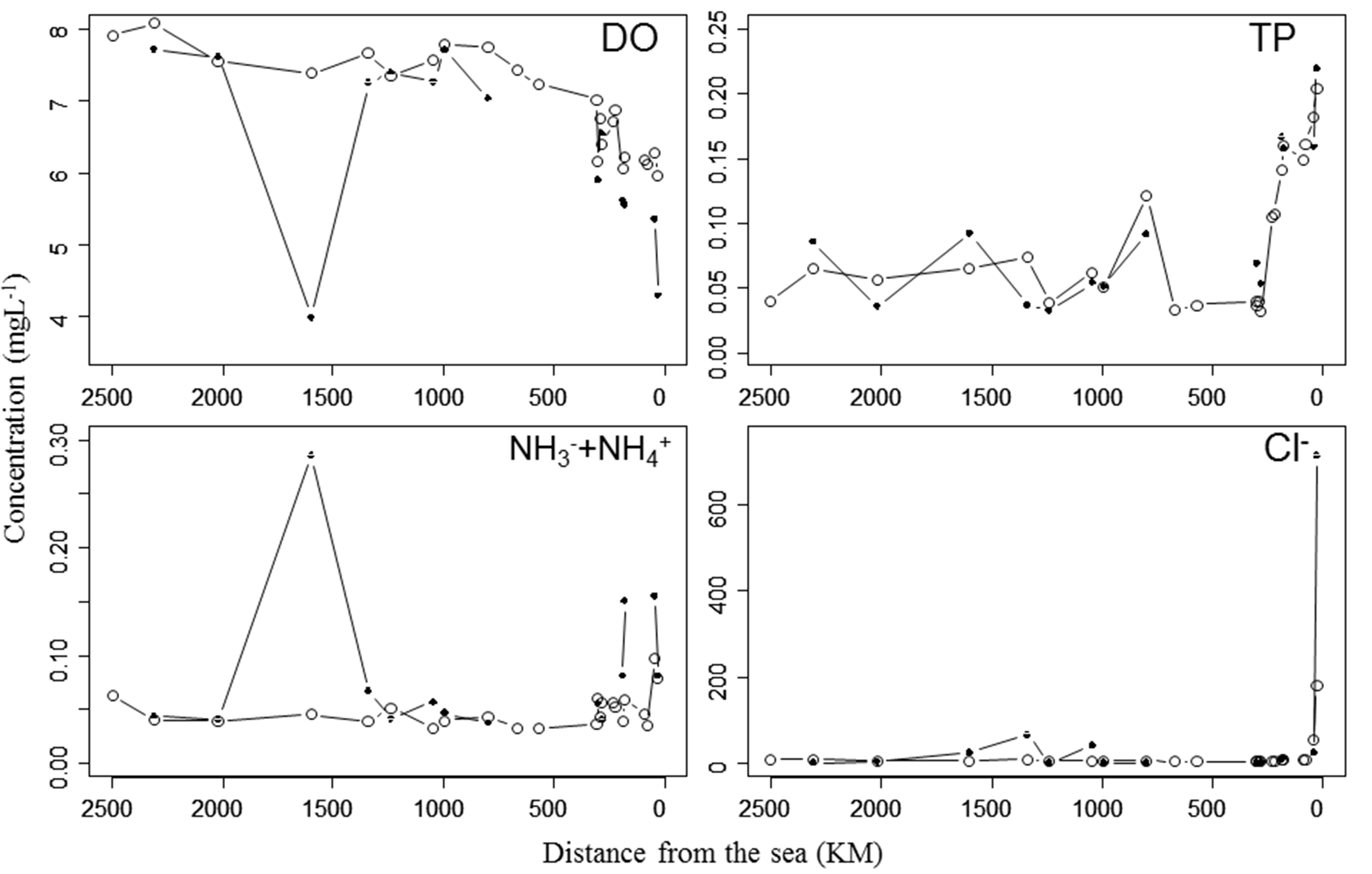

Supplement: S1 Fig — Vertical axis indicates the annual median values of concentration of DO, TP, total ammonia and Cl- in logarithmic scale (mgL-1). (TIF) [file pone.0145527.s001.tif]
